# Agraphia of *Kanji* (Chinese characters): an early symptom of sporadic Creutzfeldt-Jakob disease in a Japanese patient: a case report

**DOI:** 10.1186/1752-1947-8-269

**Published:** 2014-08-06

**Authors:** Keiko Nakamura, Kenji Sakai, Miharu Samuraki, Ichiro Nozaki, Masako Notoya, Masahito Yamada

**Affiliations:** 1 Department of Neurology and Neurobiology of Aging, Kanazawa University Graduate School of Medical Sciences, 13-1 Takara-machi, Kanazawa, Ishikawa 9208640, Japan; 2 Department of Neurology, Noto General Hospital, A-6-4 Fujihashi-machi, Nanao, Ishikawa 9260816, Japan; 3 School of Health Science, College of Medical, Pharmaceutical and Health Sciences, Kanazawa University, 5-11-80 Kodatsuno, Kanazawa, Ishikawa 9200942, Japan

**Keywords:** Agraphia, Creutzfeldt-Jakob disease, *Kana* (Japanese syllabary), *Kanji* (Chinese characters), Magnetic resonance imaging

## Abstract

**Introduction:**

Slowly progressive cognitive decline is the most frequent initial manifestation in MM2-cortical-type sporadic Creutzfeldt-Jakob disease. Agraphia has never been noted in patients with this type of sporadic Creutzfeldt-Jakob disease, however, we report the case of a Japanese patient with sporadic Creutzfeldt-Jakob disease in whom agraphia of *Kanji* was an initial cardinal symptom.

**Case presentation:**

A 59-year-old right-handed Japanese woman complained of agraphia of *Kanji* (Chinese characters) as an initial symptom. A neurological examination revealed mild word-finding difficulty, constructive disturbance, hyperreflexia in her jaw and lower limbs, and bilateral extensor plantar reflexes. An examination of her cerebrospinal fluid revealed increased levels of 14-3-3 and total tau proteins, and abnormal conformation of the proteinase K-resistant prion protein. Diffusion-weighted magnetic resonance imaging showed diffuse hyperintensity in bilateral cerebral cortices. Single-photon emission computed tomography scans revealed hypoperfusion in the left temporal lobe, bilateral parietal and occipital lobes. An analysis of the prion protein gene demonstrated no mutation with homozygous for methionine at the codon 129. We diagnosed our patient with sporadic Creutzfeldt-Jakob disease. Although a histological examination was not performed, it was assumed that our patient could be the MM2-cortical type according to the clinical findings and the elevated levels of 14-3-3 protein in her cerebrospinal fluid. The left posterior inferior temporal area, which was affected in our patient as a hypoperfusion area, is associated with selecting and recalling *Kanji* characters.

**Conclusions:**

Focal signs as an early symptom and hypoperfusion areas in sporadic Creutzfeldt-Jakob disease are critical to recognize initial brain lesions damaged by the proteinase K-resistant prion protein accumulation.

## Introduction

Creutzfeldt-Jakob disease (CJD), a degenerative neurological disorder caused by prions, is neuropathologically characterized by the accumulation of the proteinase K-resistant prion protein (PrP^res^), which leads to spongiform changes in tissues of the central nervous system. CJD is classified according to its causes: sporadic CJD (sCJD), the idiopathic form; familial CJD, caused by inherited mutations in the prion protein (PrP) gene; and acquired CJD, related to previous infectious episodes [1]. Sporadic CJD is classified into six types based on the genotype at polymorphic codon 129 of the PrP gene and the physicochemical properties of the pathologic PrP^res^: MM1, MM2, MV1, MV2, VV1, and VV2 [2]. MM2-type sCJD comprises of two pathological phenotypes: cortical and thalamic forms. MM2-cortical-type sCJD is the most common subtype as an atypical sCJD form in Japan [3]. Although slowly progressive cognitive decline is the most frequent initial manifestation in this subtype, aphasia, ataxia, psychiatric symptoms, and visual disturbance are also described [1,4,5]. However, agraphia has never been noted in patients with MM2-cortical-type sCJD.

The Japanese language has two writing systems, that is, *Kanji* (Chinese characters) and *Kana* (Japanese syllabary), which are different from those of Western languages. *Kanji* are the structurally complex morphograms introduced from China, often having several phonetic readings, while *Kana* are the relatively simple syllabograms having unambiguous phonetic readings [6]. Japanese sentences consist of various combinations of *Kanji* and *Kana*. The major lexical morphemes of Japanese words are written in *Kanji*, and conjugated endings of verbs, adjectives, and functional words are written in *Kana*. Both these systems are associated with distinct regions of the brain [7].

We report a Japanese patient with sCJD in whom agraphia of *Kanji* was an initial cardinal symptom. This patient was presumed to be MM2-cortical-type sCJD according to the clinical presentation.

## Case presentation

A 59-year-old right-handed Japanese woman had difficulty in writing *Kanji*. She could neither recognize forms of the *Kanji* characters clearly nor write them. One month later, she developed progressive cognitive impairment; however, her social behavior remained appropriate.

A neurological examination performed two months after the disease onset revealed mild word-finding difficulty and constructive disturbance such as copying simple diagrams. Hyperreflexia was present in her jaw and lower limbs. Her bilateral extensor plantar reflexes were positive, however, she showed no cerebellar ataxia, anopsia, myoclonus, or extrapyramidal signs. Moreover, neither ideomotor apraxia nor ideational apraxia was apparent.

The Standard Language Test of Aphasia, a standardized test for Japanese aphasic patients, performed three months after the disease onset revealed impaired dictation of *Kanji* words; however, other categories of the test were scored well, that is, dictation of *Kana* letters, pronunciation of words written in *Kanji* and *Kana*, and repetition and auditory comprehension of words and sentences. She scored 24 on the Mini-Mental State Examination with impairments in delayed recall, calculation, and copying interlocking pentagons.

A hematological examination revealed no abnormalities. An investigation of the cerebrospinal fluid (CSF) disclosed increased levels of 14-3-3 protein (616μg/mL) and total tau protein (1217pg/mL), although cell counts and protein levels were normal. Abnormal conformation of PrP^res^ was detected in the CSF by real-time quaking-induced conversion (RT-QUIC) [8].

**Figure 1:**
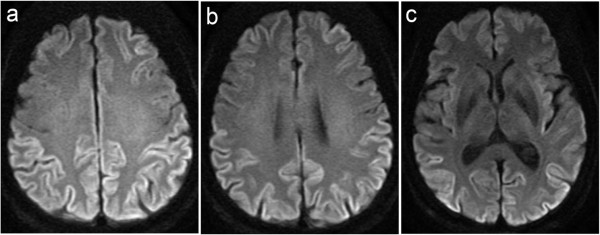
**Axial sections of the diffusion-weighted magnetic resonance images of the brain.** These images **(a-c)** reveal cortical hyperintensity in the bilateral parietal, occipital, and temporal lobes.

**Figure 2:**
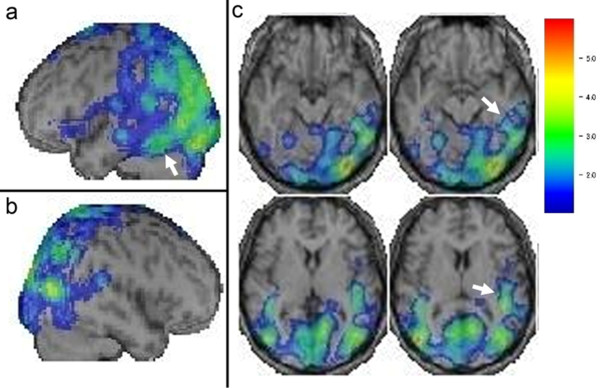
**Single-photon emission computed tomography scans evaluated using the easy z-score imaging system (a-c).** Hypoperfusion areas including the bilateral parietal and occipital lobes, left temporal lobe, and left posterior inferior temporal lobe (white arrows) are shown. **(a)** Left lateral view. **(b)** Right lateral view. **(c)** Axial views.

The electroencephalogram showed an 8 to 10 Hz basic wave pattern with no periodic discharges. Diffusion-weighted imaging (DWI) on magnetic resonance imaging (MRI) showed diffuse hyperintensity in the bilateral cerebral cortices of the parietal, occipital, and temporal lobes (Figure 1). Single-photon emission computed tomography (SPECT) scans, evaluated using the easy z-score imaging system, displayed hypoperfusion in the bilateral parietal and occipital lobes, the left temporal lobe, and in the left posterior inferior temporal lobe (Figure 2). No mutations were detected in the open reading frame of the PrP gene, and polymorphisms at codons 129 and 219 were homozygous for methionine and glutamine, respectively. Although our patient did not meet the World Health Organization (WHO) clinical diagnostic criteria for sCJD, we clinically diagnosed her with sCJD supposedly an MM2-cortical type, based on the MRI findings, elevation of 14-3-3 and tau protein levels in the CSF, and a positive result upon RT-QUIC [4,8].

Although her cognitive decline progressed, she had lived more than two years after the disease onset.

## Discussion

This is the first report of agraphia of *Kanji* as a cardinal manifestation in a patient with sCJD supposedly an MM2-cortical type. This subtype of CJD is characterized by hyperintensity regions in the cerebral cortices and/or basal ganglia on brain DWI, increased levels of 14-3-3 protein in the CSF, the rare presence of periodic discharges on electroencephalogram, and a slowly progressive clinical course [1,4]. Patients with this form do not always meet the current WHO diagnostic criteria in the early stage [4].

Our patient showed agraphia of *Kanji* exclusively at the onset of the disease, with preservation of other Japanese language abilities. Although agraphia of *Kanji* has been reported to signify damage to the posterior inferior temporal lobe, inferior parietal lobule, superior parietal lobule, or posterior middle frontal gyrus in the dominant hemisphere, the left posterior inferior temporal cortex is associated with the fundamental mechanisms of *Kanji* writing [9], that is lexical-orthographic processing, selecting the correct *Kanji* graphemes against the meanings of words and recalling the visual engrams of the characters [7]. Although the brain DWI revealed widespread hyperintensity areas in our patient, the hypoperfusion area seen in the left posterior inferior temporal lobe (Figure 2) is likely to be related to agraphia of *Kanji*.

Details of the pathomechanisms of sCJD are still uncertain. According to the protein propagation theory, PrP^res^ is created in one brain cell due to the failure of the quality control complex of proteins. Then, aggregation and replication of PrP^res^ by template conversion of normal PrP could occur. The formed PrP^res^ could propagate to other regions of the central nervous system [1]. Regarding this hypothesis, little evidence of initial lesions in patients with sCJD is established due to difficulty of obtaining neuropathological evidence in their early stages. Further assessments of the relationship between the early symptoms of patients with sCJD and the results of the functional images are crucial to clarify PrP^res^ initiation and propagation in human brain.

## Conclusions

We report a first case of sCJD with agraphia of *Kanji* as an initial and cardinal symptom. It is assumed that this patient could be categorized as MM2-cortical type according to the clinical presentation. Focal signs as an early symptom and functional imaging in early-stage sCJD are critical to recognize initial brain lesions damaged by PrP^res^ accumulation and subsequent abnormal protein propagation.

## Consent

Written informed consent was obtained from the patient and the patient’s next of kin for publication of this case report with any accompanying images. A copy of the written consent is available for review by the Editor-in-Chief of the journal.

## Abbreviations

CJD: Creutzfeldt-Jakob disease; CSF: cerebrospinal fluid; DWI: diffusion-weighted imaging; MRI: magnetic resonance imaging; PrP: prion protein; PrP^res^: proteinase K-resistant prion protein; RT-QUIC: real-time quaking-induced conversion; sCJD: sporadic CJD; SPECT: single-photon emission computed tomography; WHO: World Health Organization.

## Competing interests

The authors declare that they have no competing interests.

## Authors’ contributions

KN collected the clinical data and drafted the manuscript. KS, MS, and IN were involved in critically revising the manuscript for important intellectual content. MN assessed the language dysfunction in addition to the cognitive impairment of our patient. MY is the supervising consultant and gave the final authorization for publication of the manuscript. All authors read and approved the final manuscript.

## Authors’ information

KN, KS, MS, IN and MY have enough experience to take care of patients with prion diseases. MN is competent to examine patients with aphasia.
